# Prenatal Exposure to Maternal Bereavement and Childbirths in the Offspring: A Population-Based Cohort Study

**DOI:** 10.1371/journal.pone.0103353

**Published:** 2014-07-28

**Authors:** Oleguer Plana-Ripoll, Jørn Olsen, Per Kragh Andersen, Guadalupe Gómez, Sven Cnattingius, Jiong Li

**Affiliations:** 1 Section for Epidemiology, Department of Public Health, Aarhus University, Aarhus, Denmark; 2 Department of Epidemiology, Fielding School of Public Health, University of California Los Angeles, Los Angeles, California, United States of America; 3 Department of Biostatistics, University of Copenhagen, Copenhagen, Denmark; 4 Departament d'Estadística i Investigació Operativa, Universitat Politècnica de Catalunya, Barcelona, Spain; 5 Clinical Epidemiology Unit, Department of Medicine Solna, Karolinska Institute, Stockholm, Sweden; Oslo University Hospital, Ullevål, Norway

## Abstract

**Introduction:**

The decline in birth rates is a concern in public health. Fertility is partly determined before birth by the intrauterine environment and prenatal exposure to maternal stress could, through hormonal disturbance, play a role. There has been such evidence from animal studies but not from humans. We aimed to examine the association between prenatal stress due to maternal bereavement following the death of a relative and childbirths in the offspring.

**Materials and Methods:**

This population-based cohort study included all subjects born in Denmark after 1968 and in Sweden after 1973 and follow-up started at the age of 12 years. Subjects were categorized as exposed if their mothers lost a close relative during pregnancy or the year before and unexposed otherwise. The main outcomes were age at first child and age-specific mean numbers of childbirths. Data was analyzed using Cox Proportional Hazards models stratified by gender and adjusted for several covariates. Subanalyses were performed considering the type of relative deceased and timing of bereavement.

**Results:**

A total of 4,121,596 subjects were followed-up until up to 41 years of age. Of these subjects, 93,635 (2.3%) were exposed and 981,989 (23.8%) had at least one child during follow-up time. Compared to unexposed, the hazard ratio (HR) [95% confidence interval] of having at least one child for exposed males and females were 0.98 [0.96–1.01] and 1.01 [0.98–1.03], respectively. We found a slightly reduced probability of having children in females born to mothers who lost a parent with HR = 0.97 [0.94–0.99] and increased probability in females born to mothers who lost another child (HR = 1.09 [1.04–1.14]), the spouse (HR = 1.29 [1.12–1.48]) or a sibling (HR = 1.13 [1.01–1.27]).

**Conclusions:**

Our results suggested no overall association between prenatal exposure to maternal stress and having a child in early adulthood but a longer time of follow-up is necessary in order to reach a firmer conclusion.

## Introduction

Almost all European and East Asian countries have witnessed a decline in birth rates to low or very low levels [1], primarily explained by changes in socio-economic factors and longer education for females, resulting in a delayed start of reproduction [2] or a wish to have fewer children [3]. Fertility is therefore related to cultural and social factors as well as fecundity and it is partly determined before birth by the intrauterine environment [3]. A woman's fertility is determined by the number and maturity of her ovarian follicles before birth, and normal fetal follicular development depends on the mother's diet and other lifestyle factors. Similarly, adult male sperm production is determined largely by the development of sperm-nurturing Sertoli cells in the embryonic testes and is probably dependent on prenatal exposure to sex hormones, which again is influenced by mother's lifestyle and other environmental factors [3].

Maternal stress is one of the environmental factors that could affect the fetus. During pregnancy, the actions of glucocorticoids are balanced between positive and negative effects [4] and an overexposure puts both the mother and fetus at risk. Evidence from experimental studies suggests that excessive stress hormones in pregnant mothers cross the placenta and enter the fetal circulation [5], [6]. In this case, the function of 11β-hydroxysteroid dehydrogenase type 2 (11β-HSD2), the primary feto-placental “barrier” to maternal hormones, could potentially be affected [7]–[10]. Increased maternal cortisol further causes a rise in placental corticotropin-releasing hormone, which activates the fetal hypothalamic-pituitary-adrenal (HPA) axis and increases the fetus' own production of stress hormone [8]–[10]. Endocrine dysregulation could thus negatively affect fetal development and potentially program future disease development [8]–[11].

There is evidence from animal studies showing that different kind of prenatal stress or exposure to synthetic glucocorticoids might be related to adult fertility impairments [12]–[19]. The limited evidence in humans reflects the difficulty in obtaining information on exposures that happened several decades ago.

We hypothesized that severe prenatal exposure to maternal stress could affect the fertility of the offspring in both males and females. We defined severe prenatal stress as being exposed to maternal bereavement caused by the death of a close relative during pregnancy, which has been classified as one of the most stressful life events one can experience [20], [21]. Using the population registers available in the Nordic countries, we also defined the familial relationship of the deceased to the mother, whether the death was expected or not and the timing of death in relation to pregnancy, exposure characteristics which have been suggested to play a role in the association [21]–[23]. As an indicator of fertility in offspring prenatally exposed or unexposed to maternal bereavement, we used the age of having the first child and the age-specific total number of children. Fertility represents not only fecundity but also other factors like family planning or economic means for having a child. We hypothesized that severe prenatal stress exposure would reduce fecundity in both males and females, leading to a longer waiting time to first childbirth assuming these other factors remain unaffected. We also hypothesized that the most severe exposure would be in subjects born to mothers who lost a child or the spouse, or unexpectedly lost a close relative.

## Materials and Methods

### Design, setting and study population

This population-based cohort study considered all subjects born in Denmark and Sweden, as described the *Nordic Perinatal Bereavement Cohort* [24]. We included all Danish children born between 1968 and 1996 and all Swedish children born between 1973 and 1994. Subjects born after these dates were not included because they were younger than 12 years of age at the end of follow-up (2008 in Denmark, 2006 in Sweden). After excluding 2 children with unknown sex and 7,165 children without linkage to the mother, 2,112,770 males and 2,008,826 females were included in the study. The unique personal identification number, which is assigned to all live-born children and new residents, was used to link children to their relatives and to obtain information on demographic variables, vital statistics, health outcomes and socio-economic variables from different national registers.

### Exposure and outcome

The exposure was defined as maternal bereavement in the prenatal period. We categorized children as exposed to prenatal stress if their mothers lost a child, a spouse, a sibling or a parent within 1 year before pregnancy or during pregnancy. The remaining children were included in the unexposed cohort. We were able to link the different relatives using the Danish Civil Registration System [25] and the Swedish Multi-generation Register [26].

We used the age in which each subject had the first child as a main outcome and we also modeled the age-specific mean numbers of childbirths comparing exposed to unexposed. The date of birth of the children was obtained from the Medical Birth Registries [27], [28]. The subjects were followed from 12 years of age until death, emigration or end of follow-up (2008 in Denmark and 2006 in Sweden), whichever came first.

### Explanatory variables

Covariates were selected *a priori* according to previous literature and a directed acyclic graph [29] in order to identify the best possible strategy to adjust for potential confounders. Age of the mother was included as a categorical variable (<27, 27–30, >30 years), maternal origin was categorized as Nordic countries or other and maternal years of education had 3 categories (Low, ≤9; Middle, 10–14; High, ≥15 years). Finally, the birth year of the subject was considered for several reasons. The study recruitment period is long and the availability of birth control methods, as well as social and cultural factors affecting the decision to have children, has changed during these decades. Moreover, it is also important to adjust for it since it is related with the exposure because the quality of the registers improves over time.

### Statistical analysis

#### Missing information

There were few missing values in the original registers (see Table 1) but a complete case analysis may still lead to biased estimates [30]. We addressed this problem by using multiple imputations, obtaining unbiased and more precise and powerful estimates, assuming data was missing at random [30], [31]. Briefly, multiple imputation makes use of known subject characteristics to create different imputed datasets, incorporating appropriate variability. Each of these datasets is then analysed and the results are combined by use of the Rubin's rule [30], [31], producing a single set of estimates. We generated 50 complete datasets with imputed data using the following variables: country, date of birth, sex, maternal age and parity, birth weight and being born preterm and singleton. We compared the results when using the complete-case analysis (including only those subjects with full information on the variables needed) with analysis based on the imputed datasets. All imputations were implemented with the *ice* add-on command, and the built-in *mi* estimate command of STATA/SE 11 (Stata Corporation, College Station, TX, USA).

**Table 1:** Baseline characteristics of the study population.

	MALES (N = 2,112,770)				FEMALES (N = 2,008,826)			
	Exposed		Unexposed		Exposed		Unexposed	
Number of subjects	47898	2.27%	2064872	97.73%	45737	2.28%	1963089	97.72%
Country								
Denmark	15346	32.04%	957085	46.35%	14771	32.30%	913246	46.52%
Sweden	32552	67.96%	1107787	53.65%	30966	67.70%	1049843	53.48%
Maternal age								
<20 years	1147	2.39%	97796	4.74%	1170	2.56%	93226	4.75%
20–24 years	9024	18.84%	561194	27.18%	8594	18.79%	534558	27.23%
25–29 years	17012	35.52%	778563	37.71%	16175	35.37%	738711	37.63%
30–34 years	13753	28.71%	451208	21.85%	13155	28.76%	428698	21.84%
> = 35 years	6962	14.54%	175887	8.52%	6642	14.52%	167682	8.54%
Missing	0	0.00%	224	0.01%	1	0.00%	214	0.01%
Maternal education*, n	45996		1603828		43933		1521677	
Low, < = 9 years	12597	27.39%	410230	25.58%	11986	27.28%	390509	25.66%
Middle, 10–14 years	24689	53.68%	856773	53.42%	23443	53.36%	812251	53.38%
High, > = 15 years	8228	17.89%	264214	16.47%	8023	18.26%	249731	16.41%
Missing	482	1.05%	72611	4.53%	481	1.09%	69186	4.55%
Maternal country of origin								
Nordic	47244	98.63%	1982697	96.02%	45140	98.69%	1884966	96.02%
Non-Nordic	632	1.32%	71477	3.46%	566	1.24%	67421	3.43%
Missing	22	0.05%	10698	0.52%	31	0.07%	10702	0.55%

All the information is in n(%).

*Available from 1981 in Denmark and 1973 in Sweden.

#### Data analysis

We analysed the data stratifying by sex of offspring. A descriptive analysis was performed using frequencies (percentages) for baseline characteristics according to exposed and unexposed groups. For the main outcome of interest (age of having the first child), incidence rates (IR) of the outcomes were calculated by person-years (for the numerator we used number of childbirths and for the denominator the total number of person-years of observation). Multiple regression analysis was performed using the Cox Proportional Hazards model in order to estimate the association between the exposure and the (age-specific) rate of having the first child. Individuals never entered the risk set if they died, were lost to follow-up or emigrated before the age of 12. Subjects who did not have any children during the observation period were censored at the time of any death, emigration or at the end of the administrative end of follow-up, whichever came first. In stratified analyses, we investigated whether the estimates were different for Denmark and Sweden. Because there is evidence suggesting higher mortality among children prenatally exposed to maternal bereavement [32], we accounted for the competing risk of death before the development of the outcome using a Fine-Gray model [33]. A competing risk event was considered when any cause of death occurred without a preceding outcome. Subanalyses were performed using Cox Proportional Hazards models categorizing the exposed children by factors including type of death (unexpected or other), familial relationship to the mother/child and timing of bereavement (categorized as preconceptional, 7–12 months before conception and 0–6 months before conception; or prenatal, 1st, 2nd and 3rd trimester). Because some studies suggest an impact of early stressors on risky behaviors, including early sexual behavior and teenage childbearing [34]–[37], we also performed a subanalysis starting the follow-up time at the age of 20 instead of 12 (including 1,340,277 males and 1,241,312 females). In order to estimate the association between exposure and age-specific mean numbers of childbirths, we used a Cox Proportional Hazards model in which subjects entered the risk set again after each childbirth [38]. The proportionality of the hazards in the Cox Proportional Hazards models was evaluated using Schoenfeld residuals and evaluating the interaction of the covariates with a function of time.

All results are expressed with 95% confidence intervals (CIs). The level of significance was considered at 1% and all the analysis were performed using STATA/SE 11 (Stata Corporation, College Station, TX, USA).

### Ethics Statement

The study was based on secondary data and all analysis done at the secure platform of Denmark Statistics using encrypted identification numbers with no access to personal identification numbers of the participants. The study was approved by the Danish Data Protection Agency (J NR. 2008-41-2680) and the local ethics committee in Central Denmark Region (VEK, case number M-201000252) and Karolinska Institutet (No. 2008/4:6).

## Results

A total of 4,121,596 children were followed-up until up to the age of 41 years (median age at end follow-up time: 22.6 years). Of these children, 93,635 (2.3%) were born to mothers who had experienced bereavement during pregnancy or the year before and, therefore, considered exposed. During the follow-up time, 424,485 males (20.1%) and 557,504 females (27.8%) in the cohort had at least one child. Among those, the median age of having the first child was 27.7 and 26.1 years for males and females, respectively. Only 11.4% of males and 22.5% of females who had at least a child were younger than 22.6 years at the birth of the first child, which was the median age at the end of follow-up time of this study. Altogether, 12,615 males (0.6%) and 5,028 females (0.3%) died without having any children.

Table 1 shows the baseline characteristics of exposed and unexposed males and females. The children in the exposed group tended to be born to mothers who were older and the percentage of exposed was higher in Sweden.

Overall, we found limited evidence of an association between prenatal stress due to maternal bereavement and the age of having the first child for both males and females (Table 2). Compared to unexposed, the hazard ratio (HR) and 95% confidence interval (CI) of having at least one child for exposed males and females were HR = 0.98, CI: 0.96–1.01 and HR = 1.01, CI: 0.98–1.03, respectively. These estimates were obtained after adjusting for birth year, country, maternal age and maternal origin. We did not adjust for maternal years of education because it was only available from 1981 in Denmark. However, a subanalysis including this adjustment (with only Swedish children and Danish children born after 1981) showed similar estimates but with wider confidence intervals (results not shown). We also performed a subanalysis stratifying by country and results did not change considerably for females (HR = 1.02, CI: 0.97–1.07 for Denmark and HR = 1.01, CI: 0.98–1.03 for Sweden). For males, estimates pointed in different directions but with overlapping confidence intervals (HR = 1.03, CI: 0.97–1.09 for Denmark and HR = 0.98, CI: 0.95–1.02 for Sweden).

**Table 2:** Hazard ratio (HR) for children exposed to stress following maternal bereavement depending on different exposure status.

	MALES			FEMALES		
	Cases	Crude HR	Adjusted HR (95% CI)*	Cases	Crude HR	Adjusted HR (95% CI)*
Unexposed	419661	1.00	1.00 (ref)	550119	1.00	1.00 (ref)
Exposed	4824	0.86	0.98 (0.96–1.01)	7385	0.86	1.01 (0.98–1.03)
Timing of bereavement						
12–7 month before conception	1326	0.89	1.02 (0.96–1.07)	1961	0.84	0.98 (0.94–1.03)
6-0 month before conception	1608	0.87	0.98 (0.93–1.02)	2574	0.90	1.03 (1.00–1.08)
First trimester	556	0.83	0.96 (0.88–1.04)	837	0.86	1.01 (0.94–1.08)
Second trimester	623	0.86	0.99 (0.92–1.07)	956	0.87	1.04 (0.97–1.11)
Third trimester	711	0.83	0.97 (0.90–1.04)	1057	0.81	0.96 (0.90–1.02)
Type of relative						
Death of spouse	119	0.99	1.02 (0.85–1.22)	199	1.26	1.29 (1.12–1.48)
Death of a child	1140	0.95	1.02 (0.96–1.08)	1724	1.01	1.09 (1.04–1.14)
Death of a parent	3358	0.83	0.97 (0.94–1.00)	5153	0.81	0.97 (0.94–0.99)
Death of a sibling	207	0.98	1.09 (0.95–1.25)	309	1.02	1.13 (1.01–1.27)
Cause of death§						
Other	4154	0.85	0.97 (0.94–1.00)	6288	0.83	0.98 (0.95–1.00)
Unexpected	662	1.00	1.08 (1.00–1.17)	1078	1.13	1.21 (1.14–1.29)

*Adjusted for birth year, country, maternal age and origin.

§ Cases with missing exposure status: 171 for Danish males and 170 for Danish females.

Results did not change considerably when using a Fine-Gray model to account for the competing event of death (results not shown). We also performed a subanalysis, including only subjects with information on all variables (case-complete analysis) and we found similar results (results not shown).

The associations between having a child and different types of bereavement, depending on the timing, the type of the deceased relative and the type of death, are shown in Table 2. We did not find strong evidence that these exposure statuses play an important role, except for females when considering kind of relative and type of death, but not for timing of bereavement, according to the results obtained from a Wald-like test (results not shown). In females born to mothers who lost a parent during pregnancy or the year before, we found a slightly reduced probability of having children (HR = 0.97, CI: 0.94–0.99). On the other hand, we found an increased probability in females born to mothers who lost another child (HR = 1.09, CI: 1.04–1.14) or who were exposed to a sudden death of a relative (HR = 1.21, CI: 1.14–1.29). We also found an increased probability of having children in females born to mothers who lost the spouse (HR = 1.29, CI: 1.12–1.48) or a sibling (HR = 1.13, CI: 1.01–1.27).

When starting the follow-up time at the age of 20 years instead of 12, the estimates of having a child for overall exposure to maternal bereavement were approximately the same as in the main analysis (HR = 0.99, CI: 0.96–1.02 for males and HR = 1.00, CI: 0.97–1.02 for females).

**Figure 1:**
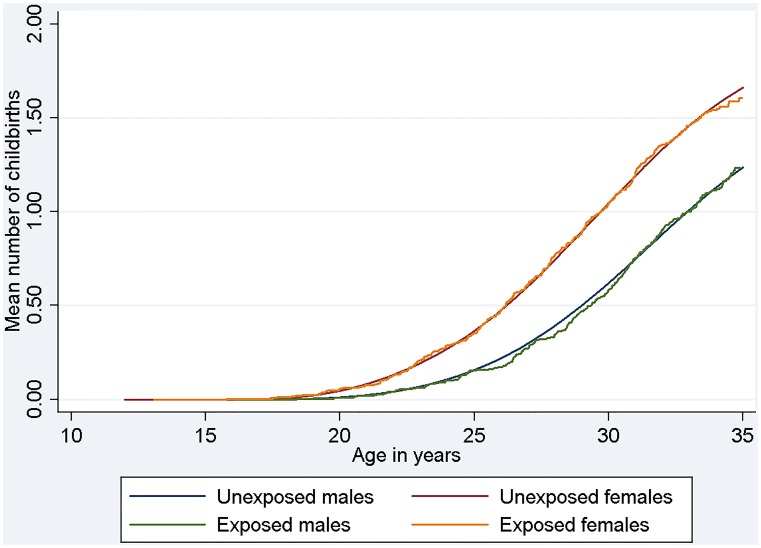
Age-specific mean number of births for exposed and unexposed males and females. Age-specific mean number of births for exposed and unexposed males and females born in Denmark between 1970 and 1975 from a mother of age <27 years and origin in the Nordic countries.

We also found an equal age-specific mean numbers of childbirths in exposed and unexposed. As a way of example, Figure 1 shows the Nelson-Aalen estimates for the mean number of childbirths depending on age for specific covariates (subjects born in Denmark between 1970 and 1975 from a mother of age <27 years and origin in the Nordic countries). The estimates from the multivariate analyses with all subjects showed no differences between exposed and unexposed (HR = 0.99, CI: 0.96–1.01 for males and HR = 1.00, CI: 0.98–1.02 for females).

We found proportionality of the hazards between exposed and unexposed in all our models. However, when evaluating the proportionality of the hazards for other covariates, we found evidence to reject the proportional hazards hypothesis for all the covariates except for maternal origin (p-value<0.001 in all the cases, results not shown) but the estimate of the coefficient of the exposure did not change considerably when including the interaction between the different covariates and the logarithm of time (results not shown).

## Discussion

In this study we found no indication to support our hypothesis that being exposed to prenatal stress is related to the probability of having the first child and the mean number of childbirths. In subanalyses, we found a slightly lower probability of having children in females born to mothers who lost a parent during the prenatal period, but higher among females born to mothers who lost another child, the spouse or a sibling. Although multiple testing could explain these few statistically significant findings, there are some theories that suggest an impact of early stressors or father absence on early sexual activity and teenage pregnancy [34]–[37]. In this case, the potential effect on having a child of prenatal exposure to maternal stress due to bereavement could be counterbalanced by the behavior of the offspring. However, a subanalysis with a follow-up starting at age 20 showed the same results as in the main analysis. The results were similar when accounting for the competing event of death before the end of follow-up without having any children, probably because the cohort was still young at the end of follow-up. Less than 1% died before end of follow-up. Results were also consistent over calendar time with birth rates being similar among exposed and unexposed.

### Strengths and limitations

This is the first study to examine if exposure to severe prenatal stress is related to the age of having a child or the number of childbirths. The main methodological strength of the study is the use of large, longitudinal, population-based registers from Denmark and Sweden with almost complete follow-up, allowing us to perform analyses with a large sample size without selection bias. The information on demographic variables is very accurate and we have therefore no information bias related to the outcomes of interest, although the data was not collected for research purposes. We believe that our measurement of exposure, bereavement following the death of a close relative, is a valid measurement for maternal stress since it is considered one of the most stressful life events [21]. It should be considered that grief or yearning are also part of the exposure but bereavement of this type is believed to cause stress irrespective of the coping style [39]. There is evidence from previous epidemiological studies that bereavement by the death of a close relative has an impact on health and mortality in widowed populations and in bereaved parents [40]–[42]. With regards to prenatal stress due to bereavement for the death of a close relative, it has been indicated that children born to mothers who lost a relative during pregnancy have an increased risk of some types of cancer [43], Type-2 Diabetes [44], childhood overweight [45], attention-deficit/hyperactivity disorder [46] and oral cleft [47].

One of the main limitations of this study is that adjusting for lifestyle factors or measuring fecundity is not possible using registry-based information. However, if exposure causes subfecundity and is not affecting family planning, subfecundity would lead to a higher age of starting procreation. We therefore decided to use the age in which exposed and unexposed subjects had their first child. Fertility represents not only fecundity but also cohabitation, a wish for a given family size or having economic means for having a child. The results could therefore be counterbalanced if these conditions differ between exposed and unexposed. It is therefore not possible to make strong conclusions from this study regarding biological fertility (fecundity) or other factors affecting fertility. We also have to consider that fecundity could be affected by chronic diseases induced by prenatal stress. If this was the case, these chronic diseases would have mediated the relationship between prenatal stress and fertility. The advantage of using childbirths to measure the outcome is that we can use existing registries of high quality and perform a population-based study. The oldest subjects of the cohort were, however, only followed-up until the age of 41 and this do not cover their full fertility life.

There were fewer missing values for some covariates in the exposed group compared with the unexposed group. This may partly be related to a higher proportion of subjects with Nordic maternal origin in the exposed group (98.7% compared with 95.8%), as information for mothers of non-Nordic origin was less complete. This may also be related to the incomplete linkage of mothers to their relatives in early years in Denmark (where there is more missing information) and, therefore, being difficult to establish exposure status. In Sweden, the proportion of exposed subjects was stable around 3% in all the periods. In Denmark, the proportion of exposed subjects increased with time due to a lack of familial linkage in the registers during the first years and, therefore, no records of the death of close relatives, indicating a misclassification of exposure status in the first years of follow-up for the oldest generation. We therefore adjusted for calendar year and performed stratified analyses by country, but obtained similar results. A subanalysis, with only subjects born after 1981, showed similar results as the main analysis. It is also important to consider that this misclassification, as well as the people who were classified as unexposed but suffered other causes of prenatal stress (for example divorce, moving or the death of a close friend), would probably draw risk estimates toward the null since they are expected to be equal frequent in the two groups.

### Comparison with existing literature

Evidence from animal studies suggests that prenatal stress could impair fertility. Ward concluded that prenatal stress feminized and demasculinized the behavior of male rats [13] while Herrenkohl showed that prenatally stressed female rats experienced fewer conceptions or more spontaneous abortions [14]. More recent studies showed that male rats prenatally exposed to stress showed reduced testosterone levels, delayed latency to the first mount or first intromission, and also decreased number of ejaculations [17]. Similarly, sexual behavior of prenatally stressed male birds is reported to be impaired [18]. These effects will be more difficult to identify in humans due to much lower fertility and, if they hold, they can be counterbalanced by a desire for a large family size, less adequate use of contraception or infertility treatment.

Another possible hypothesis is that prenatal stress changes the response to stress in the offspring in later life. Evidence has shown a relation between prenatal anxiety and differences in basal salivary cortisol levels in children [48]. Another study in adults showed that those who suffered prenatal stress, due to maternal bereavement by the death or severe illness of a relative, exhibited a higher cortisol response to psychosocial stress test than those in the control group [49].

Our results could be consistent with a study in female rats that showed that prenatal stress led to impairments of the activity of the sexual system in older ages [12]. A longer time of follow-up would be necessary to study an effect on higher age. It is also important to consider paternal behavior in the postnatal period because there appears to be a strong influence of postnatal environment on prenatal conditions where parents can reduce or reverse the effects of prenatal stress [4]. It has been shown in rats that postnatal stimulation significantly reversed the effects of prenatal stress [50].

### Conclusions

With this study, we add a large-scale human cohort study to the body of literature on prenatal stress and fertility impairments in animals. Our results suggested no overall association between prenatal stress and having a child in early adulthood. However, a longer time of follow-up is necessary in order to reach a more firm conclusion, by observing the complete fertility life of the population subjects and being able to analyse this association in older ages. It is also necessary to study fecundity more directly, and for example study menstrual disorders, sperm quality, waiting time-to-pregnancy or also considering medical causes of infertility or fertility treatments.
