# Correction: Prenatal Exposure to Maternal Bereavement and Childbirths in the Offspring: A Population-Based Cohort Study

**DOI:** 10.1371/journal.pone.0132648

**Published:** 2015-07-02

**Authors:** Oleguer Plana-Ripoll, Jørn Olsen, Per Kragh Andersen, Guadalupe Gómez, Sven Cnattingius, Jiong Li

We recently published a study on the effect of prenatal stress exposure and the offspring probability of having children [1]. We found no overall effect of prenatal exposure to maternal bereavement on the offspring probability of having a child and the number of childbirths. Surprisingly, we found that offspring prenatally exposed to maternal bereavement due to loss of an older child had a higher probability of having a child compared with unexposed offspring. The authors wish to acknowledge that not adjusting for maternal parity could have led to biased estimates. In subjects with many older siblings, the likelihood of being exposed is higher than for subjects with fewer older siblings, as more siblings are at risk of death. Due to the intergenerational transmission of fertility [2], subjects born from large families are also more likely to have children at an earlier age than subjects from smaller families and are more likely to have a larger number of children. Following the same explanation, not adjusting for the number of siblings of the mother could also lead to biased estimates for subjects prenatally exposed to the death of a mother’s sibling.

Not adjusting for maternal parity, we found in the original manuscript (Table 2) no overall effect of maternal bereavement (due to loss of a spouse, older child, parent or sibling) on the probability in offspring of having a child (boys HR = 0.98, CI: 0.96–1.01; girls HR = 1.01, CI: 0.98–1.03). We also reported a tendency towards an increased probability of having children in boys (HR = 1.02, CI: 0.96–1.08) and even stronger effect in girls (HR = 1.09, CI: 1.04–1.14) prenatally exposed to the death of an older child.

In Table 1 below, we show the same estimates shown in Table 2 of the original manuscript after further adjusting for maternal number of previous children and maternal number of siblings (the number of cases and crude estimates are not shown, as they are the same as in the original manuscript). After these adjustments, we found that both males and females exposed to maternal bereavement had an overall reduced probability of having a child (boys HR = 0.95, CI: 0.92–0.97; girls HR = 0.96, CI: 0.93–0.98). We also found a reduced probability of having a child in offspring prenatally exposed to maternal bereavement due to loss of an older child (boys HR = 0.89, CI: 0.84–0.94; girls HR = 0.92, CI: 0.88–0.96). On the other hand, females born to mothers who lost the spouse had an increased probability of having children (HR = 1.21, CI: 1.05–1.39) although this effect disappears in a subanalysis starting the follow-up at age 25 years (HR = 1.00, CI: 0.82–1.23). We also found that exposed children had a lower age-specific mean number of childbirths compared to unexposed (boys HR = 0.95, CI: 0.93–0.98; girls HR = 0.97, CI: 0.95–0.99).

**Table 1:** Hazard Ratios and 95% Confidence Intervals for children exposed to stress following maternal bereavement depending on different exposure status. Estimates adjusted for birth year, country, maternal number of older children and siblings, maternal age and origin.

	MALES	FEMALES
Unexposed	1.00 (ref)	1.00 (ref)
Exposed	0.95 (0.92–0.97)	0.96 (0.93–0.98)
Timing of bereavement		
12–7 months before conception	0.97 (0.92–1.03)	0.93 (0.89–0.97)
6–0 months before conception	0.92 (0.87–0.96)	0.96 (0.92–1.00)
First trimester	0.94 (0.86–1.02)	0.98 (0.92–1.05)
Second trimester	0.98 (0.90–1.06)	1.02 (0.95–1.08)
Third trimester	0.95 (0.88–1.02)	0.93 (0.87–0.99)
Type of relative		
Death of spouse	0.99 (0.83–1.18)	1.21 (1.05–1.39)
Death of a child	0.89 (0.84–0.94)	0.92 (0.88–0.96)
Death of a parent	0.96 (0.93–0.99)	0.95 (0.93–0.98)
Death of a sibling	1.05 (0.91–1.20)	1.07 (0.96–1.20)
Cause of death§		
Other	0.94 (0.91–0.96)	0.93 (0.91–0.95)
Unexpected	1.03 (0.95–1.11)	1.14 (1.07–1.21)

^§^ Cases with missing exposure status: 171 for Danish males and 170 for Danish females

Considering this correction, there is some evidence to support our hypothesis that being exposed to prenatal stress is related to the probability of having children and the total number of childbirths one could achieve. However, it is still necessary to study fecundity more directly in order to understand if there is a biological background for this association or these results are a result of changes in family structure induced by the exposure or other sociological changes.
